# HLA-Cw Allele Frequency in Definite Meniere’s Disease Compared to Probable Meniere’s Disease and Healthy Controls in an Iranian Sample

**Published:** 2016-07

**Authors:** Sasan Dabiri, Fatemeh Ghadimi, Mohammadreza Firouzifar, Nasrin Yazdani, Mahsa Mohammad-Amoli, Varasteh Vakili, Zahra Mahvi

**Affiliations:** 1 *Otorhinolaryngology Research Center, AmirAlam Hospital, Tehran University of Medical Sciences, Tehran, Iran.*; 2 *Endocrinology and Metabolism Research Center, Tehran University of Medical Sciences, Tehran, Iran.*

**Keywords:** HLA-Cw, Immunogenetics, Meniere’s disease

## Abstract

**Introduction:**

Several lines of evidence support the contribution of autoimmune mechanisms in the pathogenesis of Meniere’s disease. The aim of this study was determining the association between HLA-Cw Alleles in patients with definite Meniere’s disease and patients with probable Meniere’s disease and a control group.

**Materials and Methods:**

HLA-Cw genotyping was performed in 23 patients with definite Meniere’s disease, 24 with probable Meniere’s disease, and 91 healthy normal subjects, using sequence specific primers polymerase chain reaction technique. The statistical analysis was performed using stata 8 software.

**Results:**

There was a significant association between HLA-Cw*04 and HLA-Cw*16 in both definite and probable Meniere’s disease compared to normal healthy controls. We observed a significant difference in HLA-Cw*12 frequencies between patients with definite Meniere’s disease compared to patients with probable Meniere’s disease (P=0.04). The frequency of HLA-Cw*18 is significantly higher in healthy controls (P=0.002).

**Conclusion:**

Our findings support the rule of HLA-Cw Alleles in both definite and probable Meniere’s disease. In addition, differences in HLA-Cw*12 frequency in definite and probable Meniere’s disease in our study’s population might indicate distinct immune and inflammatory mechanisms involved in each condition.

## Introduction

Meniere’s disease is defined as fluctuating neurosensory hearing loss, tinnitus, vertigo, and aural pressure. The American academy of otolaryngology – Head and neck surgery (AAO-HNS) has classified Meniere’s disease based on the type and severity of symptoms. Definite Meniere’s disease is defined as two or more definitive episodes of vertigo accompanied by hearing loss, tinnitus, and/or aural fullness. Probable Meniere’s disease is defined as one definitive episode of vertigo which might be accompanied by other symptoms and signs of Meniere’s disease except for associated hearing loss (1).

A group of genetic and environmental factors are known to be responsible in the aetiology of Meniere’s disease (2,3). Also an autoimmune theory for this disease is developed (4). Major histocompatibility complexes (MHC) present pathogen-derived peptides to T cells and initiate the process of immune responses (5). A group of MHC genes are linked to autoimmune disease pathogenesis (6). Genetic studies have shown that particular HLA Alleles increase the risk of a number of autoimmune diseases (7,8). Genetic factors involved in autoimmune conditions seem to play an important role in the development of a number of inner ear diseases including neurosensory hearing loss and Meniere’s disease (9-12).

Results of our previous study, revealed that HLA-Cw is a genetic predisposing factor in definite Meniere’s disease in Iranian patients (13). In the present study we analysed the frequency of HLA-Cw alleles in patients with definite Meniere’s disease compared to those with probable Meniere’s disease versus normal controls to determine if there is any possible relation between the severity of the disease and HLA-Cw presence.

## Materials and Methods

Type of study: descriptive analytical study using a control group.

*Patients*

Twenty three patients (13 males and 10 females) with definite Meniere’s disease and twenty four patients (17males and7 females) with probable Meniere’s were selected among patients referred to the outpatient clinic of Amir-Aalam hospital between the years 2012 and 2015. Ninety-one healthy controls were selected from normal healthy subjects from the same region. The control group underwent history analysis and a physical exam to confirm their health status. We matched our control group for sex and age with both groups. Audiological, vestibular, and functional status in each patient was evaluated based on the American Academy of Otolaryngology-Head and Neck Surgery guidelines (14). The diagnosis was confirmed in all patients by ruling out the presence of anatomic lesions using magnetic resonance imaging (MRI). All the participants were requested to sign written informed consent. Patients didn’t pay for the complementary tests and their identity remained hidden throughout the study.

*HLA typing*

After taking 5cc of blood from each patient, samples were collected in EDTA tubes with anticoagulating agent stored at -20*C. The DNA was extracted using salting out method and HLA-Cw typing was performed using sequence specific primers (SSP) polymerase chain reaction [DynalAllset+TM SSP Kit] (15). 

*Statistical analysis*

HLA-Cw Allele frequencies were calculated for each group and were compared using the pearson’s chi-square test and fischer exact test. The significance of association between HLA-Cw Allele and Meniere’s disease was estimated by odds ratio (OR) and 95% confidence intervals (95% CI) using the stata software (version8). P values less than 0.05 were considered to be statistically significant.

## Results

The clinical features of the patients are shown in table 1. HLA-Cw allele frequencies were determined in all patients. The distributions of HLA-Cw frequencies were compared among different groups. There was a statistically significant difference in the frequency of HLA-Cw*04 in patients with definite or probable Meniere’s disease (18%) and healthy controls (1%) (P=0.001) (Table.2).

**Table1:** Clinical characteristics of the patients

		**Definite MD** *	**Probable MD**	**Control**	
Sex (M/F)		13/10	17/7		51/40
Mean age		34.96±13.047 (13-71)	41.04±16.815 (15-76)	38.53±10.513(14-78)	
Onset time (Years)		3.74±3.107 (1-12)	7.63±7.033 (1-24)		N/A
Unilateral/bilateral		21/1	19/5		N/A
Level of hearing	Mild	13	9		N/A
	Moderate	8	5		N/A
	Severe	2	9		N/A
Total number of patients		23	24		91

* Meniere’s Disease

**Table 2:** Distribution of HLA-Cw Alleles in patients and controls

**HLA-C allele**	**MD** * ** patients (n=47) (%) #**	**Control group (n=91) (%) #**	**P value**	**OR (CI 95%)**
Cw*01	2 (2.1%)	7 (3.8%)	0.72	0.543(0.111-2.669)
Cw*02	0 (0%)	7 (3.8%)	0.09	
Cw*03	2 (2.1%)	9 (4.9%)	0.34	0.418(0.88-1.975)
Cw*04	17 (18.1%)	2 (1.1%)	<0.0001	19.870(4.481-88.101)
Cw*06	10 (10.6%)	33 (18.1%)	0.10	0.538(0.252-1.145)
Cw*07	13 (13.8%)	25 (13.7%)	0.98	1.008(0.490-2.074)
Cw*08	4(4.3%)	4(2.2%)	0.45	1.978(0.483-8.092)
Cw*12	8(19.1%)	43(23.6%)	0.39	0.766(0.413-1.419)
Cw*13	0 (0%)	3(1.6%)	0.55	
Cw*14	7(7.4%)	17(9.3%)	0.59	0.781(0.312-1.955)
Cw*15	1(1.1%)	0(0%)	0.34	
Cw*16	17(18.1%)	9(4.9%)	<0.0001	4.244(1.811-9.943)
Cw*17	3(3.2%)	7(3.8%)	1.00	0.824(0.208-3.263)
Cw*18	0(0%)	16(8.8%)	0.002	
Total	94(100%)	182(100%)		

* Meniere’s disease

HLA-Cw*16 allele frequency was also significantly higher in patients with definite or probable Meniere’s disease compared to the controls. [p: 0.0001, OR: 4.244, 95% CI(1.81-9.94)] (Table.2).

On the other hand, HLA-Cw *18 allele frequency was significantly decreased in patients compared to the control group (P=0.002) (Table. 2).

Frequencies of HLA-Cw alleles showed significant differences between probable Meniere’s disease and definite Meniere’s disease group. There was an increase in the frequency of HLA-Cw*12 in probable Meniere’s disease group compared to definite Meniere’s disease group (P=0.046, OR; 0.328, 95% CI (0.107- 1.012)] (Table. 3). 

**Table 3:** Distribution of HLA-Cw Alleles in definite MD, probable MD patients, and controls

**HLA-C allele **	**Definite MD** * ** patients (n=23) (%)** #	**Probable MD** * ** patients (n=24) (%)** #	**Control group (n=91) (%) ** #	**P value** **(Definite vs. probable)**	**P value** **(Definit vs. Control)**	**P value** **(probable vs. Control)**
Cw*01	2 (4.3%)	0(0%)	7 (3.8%)	0.23	>0.99	0.35
Cw*02	0(0%)	0(0%)	7 (3.8%)	N/A	0.35ǂ	0.35
Cw*03	1 (2.2%)	1(2.1%)	9 (4.9%)	>0.99ǂ	0.69ǂ	0.69ǂ
Cw*04	8(17.4%)	9(18.8%)	2 (1.1%)	0.86	10^-4^	10^-4^
Cw*06	7(15.2%)	3(6.3%)	33 (18.1%)	0.15	0.64	0.46
Cw*07	8(17.4%)	5(10.4%)	25 (13.7%)	0.32	0.52	0.54
Cw*08	0(0%)	4(8.3%)	4(2.2%)	0.11ǂ	0.58ǂ	0.06ǂ
Cw*12	5(10.9%)	13(27.1%)	43(23.6%)	0.04	0.05	0.62
Cw*13	0(0%)	0(0%)	3(1.6%)	N/A	>0.99ǂ	>0.99ǂ
Cw*14	3(6.5%)	4(8.3%)	17(9.3%)	>0.99	0.77	>0.99
Cw*15	1(2.2%)	0(0%)	0(0%)	0.48ǂ	0.48ǂ	N/A
Cw*16	9(19.6%)	8(16.7%)	9(4.9%)	0.71	0.001	>0.99
Cw*17	2(4.3%)	1(2.1%)	7(3.8%)	0.61ǂ	>0.99ǂ	0.02ǂ
Cw*18	0(0%)	0(0%)	16(8.8%)	N/A	0.04ǂ	0.06ǂ
Total	#46(100%)	#48(100%)	#182(100%)			

* Meniere’s Disease,

ǂ analyzed with fisher exact test, others are analyzed using chi square

# The number of alleles is twice the number of patients, as each person has two alleles—one maternal and one paternal.

Frequencies of HLA-Cw*04 (P<0.001) and HLA-Cw*16 (P=0.001) were found to be significantly higher in definite Meniere’s disease patients compared to the control subjects. Though, the allele frequency of HLA-Cw*18 was significantly lower in definite Meniere’s disease compared to the controls (P= 0.047) (Table. 3).

## Discussion

The frequency of HLA-Cw alleles in 3 groups of definite Meniere’s disease patients, probable Meniere’s disease patients, and healthy controls has been appraised in this study. A significant association between HLA-Cw*04 and HLA-Cw*16 alleles with both probable and definite Meniere’s disease was observed in our patients. This was in agreement with the study of Koyama and colleagues in which they found significant association between HLA-Cw4 and HLA-DRB1*1602 in a Japanese sample. (16) Khorsandi et al. also found that HLA-Cw4 is found more in Iranian definite Meniere’s disease patients (13). The frequency of HLA-Cw*18 allele was significantly higher in the control group.

HLA-Cw*12 allele frequency was significantly higher in probable Meniere’s disease group compared to the patients with definite Meniere’s. The observed association of HLA-Cw alleles in our patients suggested an autoimmune mechanism in both definite and probable Meniere’s disease. This is in agreement with Greco’s study who discussed Meniere’s autoimmunity in literature (4).

A mechanism based approach to the disease diagnosis and treatment warrants more accurate diagnostic methods and more straightforward and powerful treatment approaches. Earlier studies have identified an association between the HLA-Cw7 antigen and Meniere’s disease in a British population (17). A significant increase in the frequency of HLA-Cw7 alleles in patients with Meniere’s disease was observed compared to other inner ear diseases and healthy subjects (18). Some studies have found an association between HLA-DRB1 alleles and Meniere’s disease. However the results had not been repeated in other populations (19,20).

Yeo et al. have found that frequency of HLA-Cw*0303 and HLA-Cw*0602 alleles were significantly increased in Korean patients with MD compared to healthy controls; on the other hand, HLA-B44 and HLA-Cw*0102 were significantly decreased in their patients (19). This can support the idea of the association between probable Meniere’s disease and the HLA Alleles. In another study on 80 Mediterranean patients with bilateral Meniere’s disease, an association between HLA-DRB1 *1101 and bilateral Meniere’s disease was observed; but there was no association between HLA-Cw and bilateral Meniere’s disease (20). The number of patients with bilateral Meniere’s disease was very low in our studied population.

The discrepancies in results are probably due to the ethnic or geographic background or also might be as a result of differences in phenotypic or clinical manifestations of patients in different studies which require careful interpretation. 

To the best of our knowledge, this is the first study which compares HLA-Cw allele frequencies in definite Meniere’s disease and probable Meniere’s disease patients in an Iranian sample.

Some HLA loci are inherited as haplotype blocks. Therefore the association found between HLA-Cw and Meniere’s disease in our study might be due to an association with another unknown gene in linkage with the HLA-Cw allele, which was responsible for Meniere’s disease susceptibility.

It will be very helpful to evaluate the presence of accompanied autoimmune disorders in our patients, which has not been performed in our study. In addition, population heterogeneity is a very important factor in genetic association studies as our patients were collected from a referral clinic from various ethnic and geographic origins.

## Conclusion

In conclusion, this study confirmed the association between HLA-Cw*04 and HLA-Cw*16 alleles with both definite and probable MD. HLA-Cw*12 was found as a discriminating allele between definite and probable Meniere’s disease which requires more attention in replication studies. A protective role for HLA-Cw*18 also was proposed in this study. Further larger studies and more comprehensive studies on the role of other HLA genes in susceptibility to Meniere’s are warranted.
